# Electromagnetic Tracking System for Medical Micro Devices: A Review

**DOI:** 10.3390/mi16101175

**Published:** 2025-10-16

**Authors:** Mingshan He, Aoji Zhu, Lidong Yang

**Affiliations:** Research Institute for Advanced Manufacturing, Department of Industrial and Systems Engineering, The Hong Kong Polytechnic University, Hong Kong SAR, China; mingshan.he@polyu.edu.hk (M.H.); ao-ji.zhu@connect.polyu.hk (A.Z.)

**Keywords:** electromagnetic tracking system, minimally invasive surgery, clinical applications

## Abstract

Minimally invasive surgery (MIS) has become increasingly favored by both patients and surgeons owing to its advantages such as shortened recovery times and reduced surgical trauma. To enhance intraoperative feedback from surgical instruments while minimizing harmful radiation exposure, a wide range of electromagnetic tracking systems (EMTS) has been developed at micro scales for medical applications. This review provides a comprehensive summary of advances in the field over the past five years, with an emphasis on the working principles of EMTS, system architecture, current research progress, and clinical applications. In comparison to other review papers, this article focuses specifically on EMTS for medical micro-devices, such as robotic catheters, endoscopes, and capsule robots. Moreover, Representative research studies and commercial systems are presented along with their clinical implementations, placing greater emphasis on the translation of EMTS into medical applications. Finally, this review outlines and discusses future research directions, highlighting major challenges and potential opportunities for advancing the integration of EMTS into routine clinical workflows.

## 1. Introduction

Minimally invasive surgery (MIS) has undergone rapid development since the late 20th century, significantly transforming modern surgical practice [1,2]. Compared with conventional open surgery, MIS is more acceptable to patients due to (1) shorter recovery period, (2) fewer postoperative scars, and (3) reduced wound pain resulting from smaller incisions and less tissue trauma [3]. In the past, MIS was challenging to perform due to the lack of precise measurement modalities capable of providing necessary spatial and anatomical information to physicians [4]. Nowadays, various clinical measurement modalities are available to help physicians acquire more detailed insights into a patient’s condition for more accurate and efficient operations during MIS. Computed tomography (CT) [5], magnetic resonance imaging (MRI) [6], and ultrasound (US) [7] are widely used as pre-operative image-guided diagnostic technologies, providing essential information for treatment planning and surgical decision-making [8]. Additionally, optical coherence tomography (OCT) [9] and intravascular ultrasound (IVUS) [10] are minimally invasive measurement modalities employed to reconstruct the structure of cardiovascular branches, providing critical imaging information for physicians. However, MIS still requires more safety, accurate, and reliable surgical instruments and measurement modalities to be developed, as physicians have limited intuitive feedback like tactile and visual feedback from the operating environment, especially in neurosurgery and interventional surgery [11]. This lack of feedback also contributes to the increased complexity of MIS compared to conventional open surgery, and take longer and more specialized training for surgeons to achieve proficiency [12]. Thus, the tracking systems are a kind of essential technology to receive the feedback from the preferred targets, and they are also the essential technology used in the MIS.

Tracking systems are a kind of essential technology of a feedback system for acquiring the real-time position and orientation of surgical instruments. Currently, tracking systems based on mechanical [13], acoustic [14], optical [15], and electromagnetic technologies [16] are widely used across various clinical and research applications. Mechanical tracking systems employed a physical transmission between the reference point and the expected target point to indirectly measure the position and orientation of the target point [17]. Acoustic tracking systems use the time-of-flight of the ultrasonic waves to measure the target pose [18], and it is also widely used in underwater robotics, because ultrasonic waves can propagate efficiently over long distances in water [19]. In MIS procedures, especially in orthopaedic surgery [20], the optical tracking system is frequently employed to localize the distal tip of surgical instruments using optical cameras in conjunction with markers placed on the proximal (hand-held) part of the instruments. This system is also widely accepted in neurosurgical procedures due to its high tracking accuracy and robustness, which are well-suited to the precision demands [21]. However, the optical tracking system is inherently limited by its reliance on a direct line-of-sight between the camera and the optical markers. In contrast, the EMTS enables fast and accurate tracking performance without the need for line-of-sight, by generating and sensing within a static or dynamic electromagnetic field [22]. Moreover, it takes advantage of the size of its sensor compared to the mechanical tracking system and the tracking accuracy the acoustic tracking system. Although EMTS is susceptible to distortion from nearby metal or ferromagnetic sources [23], EMTS has been rapidly developed and increasingly employed in the field of medical micro robotics for both clinical applications and diagnostic devices [24,25]. Particularly in interventional surgery, C-arm computed CT is commonly used as an image-guided system to track minimally invasive instruments such as catheters [26]. However, the X-ray radiation emitted by C-arm CT exposes physicians to an increased risk of radiation-induced conditions, including cancer [27]. In gastrointestinal examinations, endoscopy with real-time imaging is a standard approach [28]. But patients often experience discomfort during the procedure. To address these limitations, electromagnetic technology based micro device solutions have been developed for applications such as wireless capsule endoscopy [29] and micro-catheters integrated with micro coils [30] or magnetometers.

To the best of our knowledge, a significant number of high-quality research articles related to EMTS has been published over the past five years [2], including electromagnetic sensor and system design, electromagnetic tracking algorithms, and clinical applications using commercial EMTS. Franz et al. [31] presented and reviewed the fundamental working principles, scientific advancements, and commercial systems of EMTS. Then, a comparative analysis was introduced between the optical tracking systems and EMTS, and it presented the potential and limitations of the two systems in biomedical applications [32]. And Than et al. [33] analyzed the advantages and disadvantages of the localization methods based on magnetic field and electromagnetic waves for robotic endoscopic capsules applications. Moreover, Sauer et al. [34] investigated EMTS as a tool for verifying brachytherapy treatment and advancing the clinical implementation. However, contrasted with previous review papers, this review focuses specifically on advances from the last five years. This period, it is marked by the rapid technological progress in tracking performance and miniaturization. Furthermore, unlike existing surveys, we incorporate both commercial systems and micro-scale clinical applications, offering a more comprehensive perspective on the translation of EMTS into medical applications. Therefore, this work provides an up-to-date and application-oriented overview aimed at facilitating clinical adoption and inspiring future innovation.

The motivation and novelty of this review paper are to present a comprehensive and state-of-the-art overview of the current research studies, commercial devices, and clinical case studies of EMTS. Section 2 briefly introduces the working fundamental principles of EMTS and the details of the system components constituting the EMTS in Section 3. Section 4 presents the current research process related to the EMTS in different approaches and applications. Section 5 discusses both commercial electromagnetic tracking solutions and their clinical applications, highlighting and comparing their tracking performance with research studies. The representative clinical case studies employing EMTS is presented in this section. This section also discusses the primary sources of tracking errors associated with EMTS and the assessment protocols. Finally, the discussion and conclusion are presented in Section 6 and Section 7, respectively. A systematic literature search was conducted for articles published between 2020 and August 2025 using the scientific databases IEEE Xplore, PubMed, and Google Scholar. The Specific keywords (i.e., electromagnetic tracking system, electromagnetic localization system, and electromagnetic clinical applications) related to each main topic of EMTS was used during the search process. Additionally, the patents related to EMTS technologies have been systematically searched and reviewed in this paper.

## 2. Working Principle of Electromagnetic Tracking System

**Figure 1:**
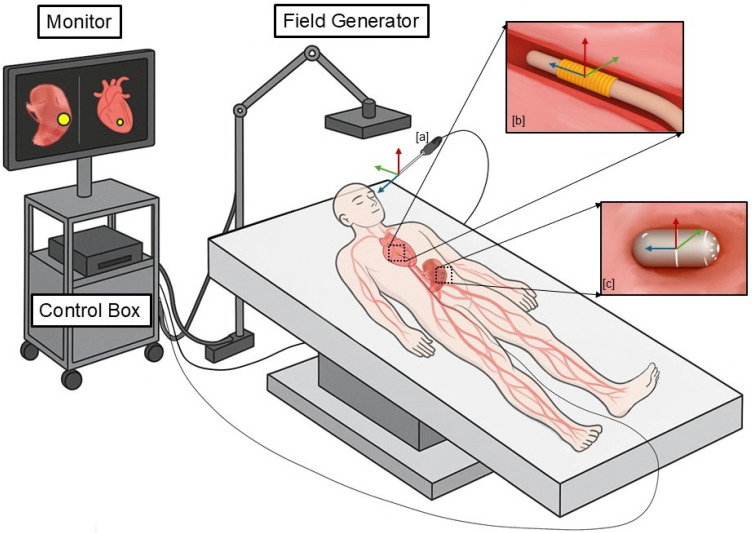
Electromagnetic tracking system. The main components include a field generator, a control box, a monitor, and a magnetic sensor used to track the (**a**) surgical instrument, the (**b**) capsule robot, or the (**c**) catheter.

EMTS can be employed in various clinical scenarios like capsule robots, catheters, and surgical instruments, as illustrated in the Figure 1.

The field generator (FG) is a controllable device capable of producing both quasi-static and dynamic magnetic fields by utilizing permanent magnets or passing electrical currents through transmit coils. Digital signal processing of sensor signals is captured and executed by circuit boards housed within the control box, which interfaces with a PC to transmit computed tracking results.

Several different types of devices could be tracked according to different sensors. For the surgical instruments, the three-dimensional EMTS is presented based on quasi-static magnetic fields and the sub-milimeter three-dimensional magnetometers integrated into surgical instruments, enabling comprehensive localization during surgical procedures [35]. And Yonggan et al. [36] proposed an induced electromotive force (EMF) prediction model based on XGBoost to correct deviations inherent in the magnetic dipole model. Then, they introduced a novel 5-DoF positioning system for micro-scales devices like catheters or guidewires by utilizing single-axis electromagnetic transmit coils to accurately locate the micro coils measuring ϕ1.45 × 5 mm according to the induction principle. Moreover, Christopher et al. [37] developed a straightforward method for identifying reference points from positional data along the trajectory of an EMT sensor and subsequently applied this technique to the capsule robot. The components of EMTS are introduced in next section.

## 3. Components of Electromagnetic Tracking System

### 3.1. Field Generators

**Figure 2:**
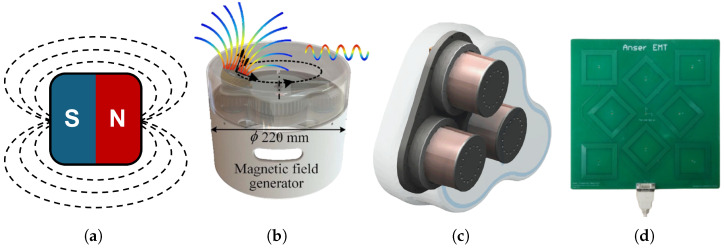
Commonly used magnetic field generators. (**a**) Permanent magnet and dotted lines mean the permanent magnetic field. (**b**) Single electromagnetic coil and color lines mean the alternative magnetic field. (**c**) Multiple electromagnetic coils. (**d**) Printed circuit board based electromagnetic coil.

A stable and reliable magnetic field is essential for the EMTS. The commonly used field generators are divided into permanent magnet and electromagnet, as shown in Figure 2. The permanent magnet could generate the quasi-static magnetic field, and the electromagnetic field could be controlled by different types of electrical current through the coils. The quasi-static magnetic field generated by the permanent magnet is commonly similar to a magnetic dipole and used to control the micro devices by changing its orientation with an external motor [38]. And in Figure 2b, the single axis of the electromangetic coil is integrated into the motor to generate a spatial differential electromagnetic field [39]. Gervasoni et al. [40] developed a three electromagnetic coil based field generator to control the micro catheters named with “Navion” as shown in Figure 2c. Moreover, a planar inductive EMTS [41] is introduced, including the magnetic field generator manufactured on the PCB [42] and embedded into medical patches as shown in Figure 2d.

Previously, the capsule robot has often carried a small permanent magnet due to its physical intensity to generate magnetic fields [43]. Since the magnetic flux intensity at the reference point originates from the permanent magnet embedded within the capsule robot, the magnetic sensor or sensor array is typically located externally to the patient’s body to determine the capsule’s position and orientation. Alternatively, transmit coils are another common choice for producing precise and stable magnetic fields in micro robotic applications [44]. The magnetic flux intensity *B* of the electromagnetic field generated by the transmit coil at the point $P_{n}$ can be expressed as [45]:

(1) $$B\left(P_{n}\right)=\frac{\mu_{0}}{4\pi }\int_{C}\frac{Idl\times r}{{\left|r\right|}^{3}}$$

where *l* is a point on path *C*, *r* is the full displacement vector from the point *l* to the point $P_{n}$, and $\mu_{0}$ is the magnetic constant.

Furthermore, the permanent magnet and transmit coils are approximately modeled as magnetic dipoles. And the magnetic flux intensity surrounding the magnetic dipole source can be represented by the following equation,

(2) $$B\left(P_{n}\right)=\frac{\mu_{0}}{4\pi }\left(\frac{3\left(\vec{m}\cdot \vec{r}\right)\vec{r}}{|\vec{r}{|}^{5}}-\frac{\vec{m}}{|\vec{r}{|}^{3}}\right)$$

where $\vec{m}$ is the magnetic dipole moment of the magnet. The permanent magnet is commonly attached on the capsule robot, which could be used as the FG for the magnetic sensor to track its magnetic field and compute its position and orientation [44]. In contrast, Gervasoni et al. [46] introduced a portable electromagnetic navigation system capable of generating a wide-area magnetic field, enabling tracking over a large workspace. Gongyue et al. [47] proposed an active EMTS based on 2-DoF magnetic source direction control. By adjusting the magnetic moment axis of the electromagnetic coil, a high-precision positioning system is achieved in all directions.

### 3.2. Sensors

Another essential component of the EMTS is the magnetic sensor. These sensors measure the magnetic field or its gradient, which varies depending on the position relative to the magnetic field source [48]. This spatial dependency is a key reason why the magnetic flux distribution around the dipole source needs to be modeled. According to Equation (2), the magnetic flux density *B* is a function of the relative position *r* between the sensor point and the magnetic field source.

**Figure 3:**
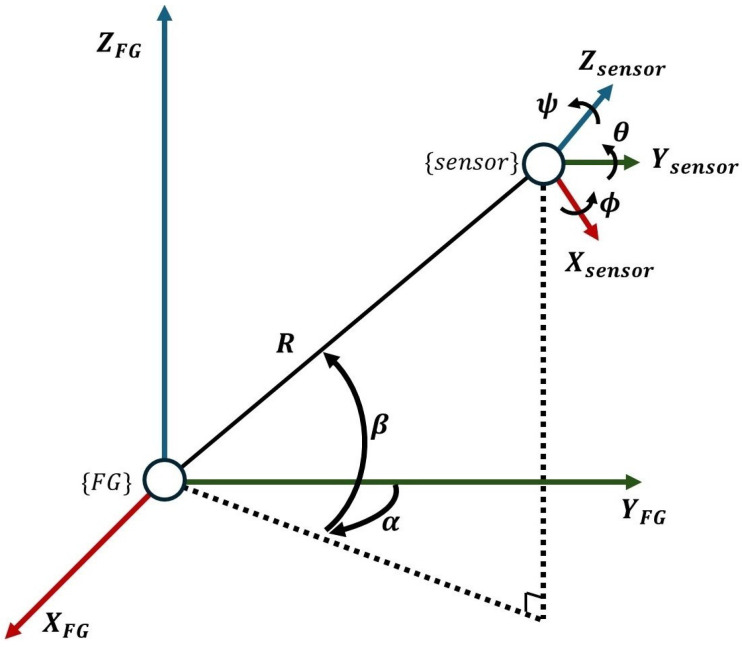
Polar coordinates for describing the tracking principle of EMTS. The field generator is approximately modeled as a magnetic dipole, with the sensor positioned in polar coordinates. The position of the sensor is represented by R,α,β, and the orientation by the RPY angle ϕ,ψ,θ.

To explain the basic tracking principle of EMTS, the polar coordinates are commonly used to describe the tracking result, as shown in Figure 3. For instance, the current through the micro coils is amplified and digitized as signals, and the signals are used to calculate the sensor’s position and orientation as a transformation from the field generator’s frame to the sensor’s frame. Therefore, by measuring the magnetic flux intensity at the sensor’s location, the spatial position and orientation of the sensor can be inferred within the electromagnetic field. The following sections provide a detailed discussion of induction principle based micro coils, integrated magnetic sensors, and magnetic field camera systems.

Micro coils utilize the principle of electromagnetic induction to measure the magnetic flux density as a function of time *t*. When exposed to an alternating magnetic field, a time-varying electromotive force (EMF) is induced in the micro coils. The induced EMF *e* can be expressed as:

(3) $$e=-N\frac{d\Phi_{B}\left(t\right)}{dt}$$

where *N* is the number of coil turns, and $\Phi_{B}$ is the magnetic flux through the coils within the magnetic field *B*. Due to the geometric limitations of micro coils, the induced EMF is typically not strong enough to be directly used for pose estimation. Therefore, amplification and filtering circuits, integrated within the control box, are required to process the induced EMF and convert it into a usable digital signal for further analysis [49]. A novel electromagnetic navigation system [50] is proposed that uses quadrature pulse-width modulation (PWM) technology to achieve remote actuation and 6-DoF pose tracking of induction coils embedded in the catheter tip. Experimental results demonstrate its suitability for minimally invasive surgery scenarios, such as navigating intravascular catheters. In addition, the material of the sensors also has an influence on their tracking performance by decreasing the distortion influence of the external source [40].

In addition to induction principles, various other types of magnetic sensors have been developed in the form of integrated circuits, such as Hall effect sensors and magneto resistive (MR) sensors [51]. The hall effect sensors are used to be integrated into the capsule robot and track its pose [52]. It also can be used to located the distal end of the surgical catheter to be localized. Moreover, simultaneous actuation and localization can be implemented with a magnetic catheter that has both a Hall effect sensor and permanent magnetic are located at the tip [53,54]. Sharma et al. [55] presented a high-resolution 3D navigation and tracking system based on CMOS technology and Hall effect sensors operating within magnetic field gradients. This system offers the potential to replace X-ray fluoroscopy in high-precision surgical procedures. And Houde et al. [56] proposed a simple and efficient electromagnetic tracking method capable of both 3-DoF and 6-DoF pose estimation. The system employs single-axis transmitting coils in combination with a three-axis anisotropic magnetoresistive (AMR) sensor.

The magnetic field camera (MFC) refers to a spatially distributed magnetic sensor array capable of capturing the three-dimensional distribution of a magnetic field source. By sampling the magnetic field simultaneously at multiple points, the magnetic field camera constructs a “magnetic image” of the environment, enabling accurate estimation of the magnetic field source through comparison with pre-calibrated field models or reconstruction algorithms [30]. This approach enhances tracking accuracy and robustness compared to single-point sensing, particularly in environments with field distortions. Recent advances have significantly improved the performance and applicability of the magnetic field camera in EMTS. The low-field magnetic field camera (MFC) is developed using high-resolution three-axis Hall effect and anisotropic magnetoresistive (AMR) snesors, achieving sub-millimeter accuracy within 21 cm and maintaining <3 mm error at 42 cm from the generator [57]. Their calibration strategy and environmental field cancellation made the system resistant to static magnetic disturbances, enabling integration into minimally invasive surgical tools. Zhang et al. [58] extended the concept of a large-workspace, eye-in-hand configuration, where a mobile magnetic field camera (MFC) mounted with actuation coils followed the target in real-time. This design not only maintained high localization accuracy (2.02 mm position RMSE, 8.24° orientation RMSE) but also introduced a signal-quality-based tracking strategy that adaptively optimized sensor placement, reducing position error by 25% compared to static arrays. In a different context, the EMTS is evaluated in the presence of a da Vinci surgical robot, demonstrating that a well-positioned magnetic field camera could sustain clinically acceptable accuracy (<1 mm, <1°) within the surgical workspace, even when large metallic structures and instruments were present [59].

**Figure 4:**
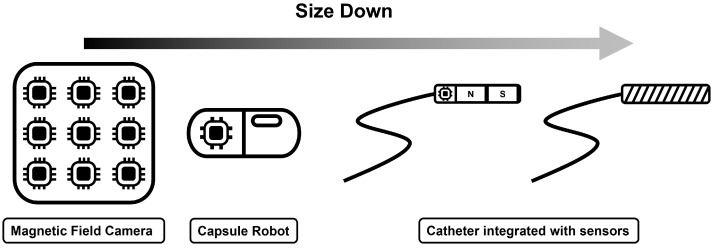
Overview of magnetic sensors from macro to micro scale: including a magnetic field camera, capsule robot, and catheters integrated with sensors. The magnetic field camera consists of multiple magnetic sensors for tracking the magnetic objects, while a single magnetic sensor can be attached to capsule robots or catheters for tracking purposes.

Overall, the mentioned magnetic sensors could be found in Figure 4, including the magnetic field camera, capsule robots, and catheter integrated with sensors. The medical applications determine the size of the sensors. As the size of the sensor is down, the cost increases. Thus, the balance of size and cost needs to be considered to take an appropriate strategy for tracking micro devices. The details of recent studies related to the mentioned EMTS will be introduced in the next section.

### 3.3. Source of Error

EMTS is subject to multiple sources of error arising from fundamental physical constraints, system design limitations, manufacturing imperfections, and environmental noise. While some errors can only be compensated for or solved at the developer level, others may be handled with careful execution and algorithm development [60,61]. In the following, these errors are classified into three principal categories: inherent system errors, field distortion errors, and motion induced errors.

Inherent system errors arise from intrinsic limitations in measurement accuracy and reproducibility of the generated magnetic field. These problems could be divided into two errors. Systematic errors can be compensated through calibration, while random errors may be mitigated by advanced filtering algorithms [62]. Field distortion errors arise from secondary, unintended magnetic fields generated by environmental ferromagnetic materials, nearby electromagnetic sources, or induced eddy currents. These external perturbations manifest as additive noise, distorting the nominal magnetic field geometry, thereby reducing localization accuracy [63]. Therefore, the compensation of field distortion is essential for enhancing system performance [64]. Motion induced errors arise from dynamic factors such as sensor velocity during acquisition and environmental changes caused by moving objects or materials. These effects can increase EM tracking errors both by introducing measurement instability and by generating transient field distortion.

Previously, the passive protection and active compensation approaches were introduced to compensate for EM errors. The passive protection is a kind of hardware solution to compensate for inherent system errors and some field distortion errors. And the active compensation is a kind of software solution. It presented several compensation algorithms to compensate for some field distortion errors and motion induced errors.

## 4. Current Research Process

**Figure 5:**
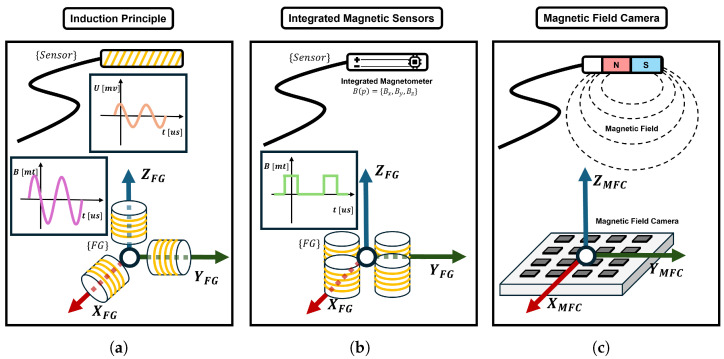
Working principles of three classic types of electromagnetic tracking systems (EMTS). (**a**) Systems based on electromagnetic induction, where a transmitter coil generates an alternating magnetic field that induces currents in receiver coils based on Faraday’s law. (**b**) Systems utilizing integrated magnetic sensors. (**c**) Systems incorporating a magnetic field camera (MFC). Integrated magnetic sensor systems and MFC both rely on compact, high-resolution sensing units from specialized manufacturers.

In this section, the current research progress on EMTS is introduced, and an overview is presented in Figure 5. The most established and widely investigated approach relies on the principle of electromagnetic induction. In this method, transmitter coils generate alternating magnetic fields, which in turn induce voltages in the receiving coils. Having been developed and commercialized for more than two decades, induction-based EMTS are now routinely available for various surgical procedures.

More recently, a second approach has emerged, based on integrated magnetic sensors (e.g., Hall-effect or anisotropic magnetoresistive sensors) combined with quasi-static magnetic fields. In this configuration, direct currents in external coils generate static or slowly varying magnetic fields, which are then measured by integrated magnetometers embedded in the target device, such as capsule endoscopes or robotic endoscopy systems. Although this method achieves comparable accuracy to induction-based tracking, its effective tracking volume and sampling rate are typically more limited.

A third approach involves the use of arrays of integrated magnetic sensors, commonly referred to as magnetic field cameras (MFCs). In these systems, a permanent magnet attached to the target device is detected by a spatially distributed sensor array, which reconstructs the three-dimensional magnetic field distribution. This method enables the tracking of a wide range of magnetic sources and allows flexible system configurations; however, the effective tracking volume remains constrained by the physical size and density of the sensor array.

### 4.1. Induction Principle

**Figure 6:**
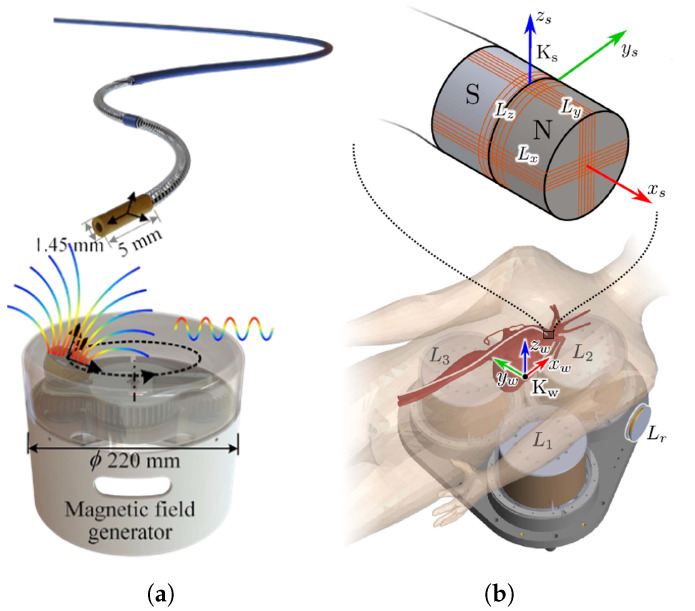
Current research studies based on the principle of electromagnetic induction. (**a**) A single-axis electromagnetic coil system developed and mounted on a motor to generate a spatially varying alternating magnetic field for tracking micro coil sensors. (**b**) Micro coil sensors integrated with permanent magnets to achieve simultaneous localization and navigation.

The fundamental working principles of EMTS have been described in the previous section. Recently, increasing attention has been focused on integrating artificial intelligence to enhance system performance. For instance, a novel EMTS was proposed that employs a single-axis electromagnetic field generation coil to localize a micro-coil, while a CNN–LSTM–based 5-DoF positioning model was designed and trained to improve tracking accuracy as Shown in Figure 6a [36]. This approach significantly reduces the number of required field generation coils and utilizes machine learning to improve tracking accuracy.

In parallel, a unified framework has been introduced for simultaneous actuation and localization of tethered magnetic devices equipped with embedded pickup coils [50]. The 6-DoF localization was achieved by driving three orthogonal electromagnets with pulse-width–modulated voltages at distinct frequencies. Demonstrated in a human-scale experimental setup, this method successfully actuated and tracked a magnetic catheter prototype with integrated pickup coils at its distal tip.

Furthermore, a custom-designed catheter incorporating a 5-DoF electromagnetic coil sensor at the tip has been developed as shown in Figure 6b [39]. The recorded sensor position was exploited not only to estimate the catheter tip orientation for navigating vessel bifurcations, but also to enable dynamic shape reconstruction as the catheter advanced or retracted within the vasculature. These advances highlight the growing role of artificial intelligence and integrated sensing–actuation strategies in extending the capabilities of EMTS beyond conventional localization.

### 4.2. Integrated Magnetic Sensors

**Figure 7:**
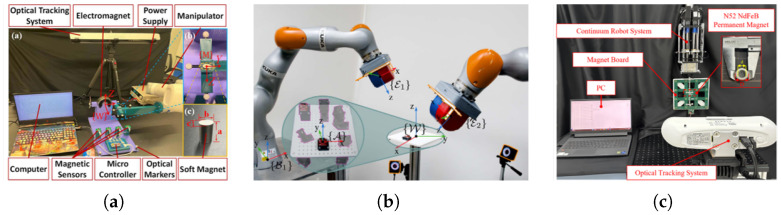
Current research based on the integrated magnetic sensors. (**a**) Development of an EMTS employing integrated magnetic sensors to track the magnetic field of a permanent magnet attached to the target device. (**b**) A new proposed algorithm for tracking a capsule robot influenced by dual external magnetic sources. (**c**) Effective tracking of end-tip trajectories of continuum robots during medical interventions using integrated magnetic sensing systems.

A wireless EMTS has been proposed for capsule endoscopy applications, as illustrated in Figure 7a. The system provides a comparative analysis of conventional “wired” EMTS and its wireless counterpart, introduces a tailored wireless EMTS algorithm, and implements a compact, low-power hardware architecture on the receiving side. This design aims to meet the stringent requirements of capsule endoscopy, including miniaturization, low energy consumption, and reliable signal transmission [65].

With the advent of new magnetic actuation systems employing multiple external permanent magnets to enhance control and manipulability, novel localization techniques are required to accommodate and exploit the additional magnetic field sources. In this context, a magnetic localization approach based on the Special Euclidean Group SE(3) has been developed, as shown in Figure 7b [66]. This method combines millimeter-scale three-dimensional accelerometers with tri-axial magnetic sensors to estimate full six-degree-of-freedom (6-DoF) pose without requiring prior initialization, thereby enabling robust localization in complex actuation environments.

The potential of continuum robots in medical interventions is considerable, owing to their flexibility and ability to navigate anatomically constrained regions. A recent framework based on a fast-adaptive permanent magnetic tracking methodology has been introduced, as depicted in Figure 7c. By incorporating a permanent magnet positioning strategy, the system achieves reliable trajectory tracking and ensures accurate navigation of continuum robots through intricate anatomical pathways [52].

### 4.3. Magnetic Field Camera

**Figure 8:**
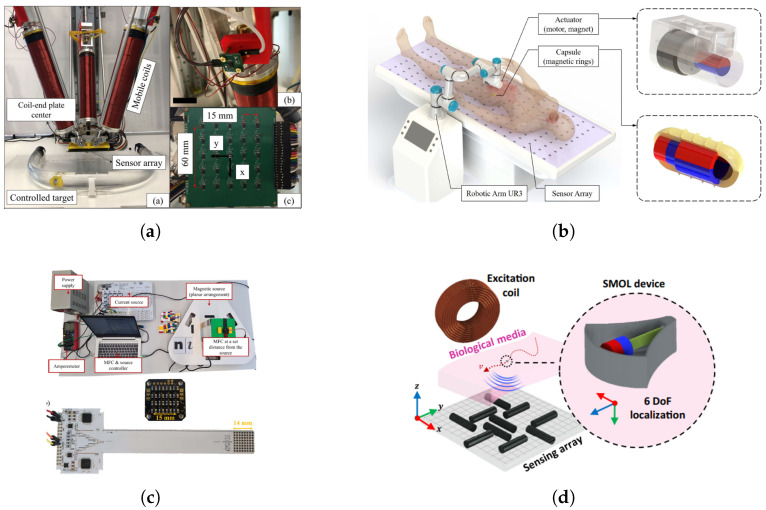
Current research studies based on the magnetic field camera. (**a**) A 5-D magnetic localization and actuation system proposed to track and actuate magnetic robots in a cylindrical workspace. (**b**) A novel system using a rotating magnetic actuator and an external sensor array to achieve closed-loop simultaneous magnetic actuation and localization for a capsule with two embedded magnetic rings. (**c**) Millimeter-sized sensors capable of measuring the local magnetic field with high resolution to sense the gradient field. (**d**) A design to track micro-scale magnets located in robots.

A 5-DoF magnetic localization and actuation system has been proposed to simultaneously track and manipulate magnetic robots within a cylindrical workspace. Leveraging an eye-in-hand sensor array configuration and the flexibility of a mobile electromagnetic manipulation platform, Zhang et al. achieved high localization accuracy using only 25 sensors across a large workspace, as shown in Figure 8a. They further developed a signal-quality–based tracking strategy, in which the movement of the sensor array is adaptively adjusted to improve localization performance [58].

Another novel system employs a rotating magnetic actuator in combination with an external sensor array to enable closed-loop, simultaneous actuation and localization of a capsule robot equipped with two embedded magnetic rings. In this approach, the capsule state is estimated by correlating the theoretical actuating magnetic field with the measured total magnetic field, thereby enabling robust closed-loop control [67], as shown in Figure 8b.

To reduce the limitations of conventional high-field EMTS, an alternative low-field EMTS has been introduced and shown in Figure 8c. This design integrates a low-power field generator with millimeter-scale, high-performance magnetic sensors capable of resolving local field gradients with high precision. Such sensors can be directly embedded into surgical tools, including catheters and deep-brain electrodes, thereby enhancing system integrability and reducing energy consumption in the operating theater [57].

Despite these advances, static magnetic localization techniques still face inherent constraints. They typically require relatively large permanent magnets and are fundamentally restricted to five degrees of freedom (5-DoF) due to the rotational symmetry around the magnetic axis. To overcome these limitations, a small-scale magneto-oscillatory localization method has been proposed, enabling wireless 6-DoF tracking of millimeter-scale devices in deep biological tissues [68], as shown in Figure 8d. This system utilizes a mechanically resonant cantilever carrying a magnetic dipole, which generates temporal oscillations to break the axial symmetry. By exploiting frequency-response characteristics, the device achieves a high signal-to-noise ratio with sub-millimeter accuracy, long-range operability, and quasi-continuous refresh rates.

## 5. Commercial Device and Clinical Application

The clinical application is the most interesting targeting field for the company to develop appropriate EMTS. Based on the modern medical modarities, the peformance of the devices can be improved to combined with EMTS for sensor fusion [69,70,71]. The commercial devices and the clinical cases are introduced in the following sections. The tracking performance of commercial devices and part of the listed studies is evaluated according to the tracking performance, like positional accuracy and orientational accuracy.

### 5.1. Commercial Device

Commercial EMTS for clinical application can be categorized into induction principle based micro coils systems and integrated magnetic sensors [72,73]. Micro coils systems employ miniature wound coils as sensing components, enabling high-precision pose tracking. It is represented by the Northern Digital Inc. (NDI) [74] Aurora platform and 3D Guidance platform. Such devices can be embedded into guidewires, biopsy needles, or catheters, and maintain sub-millimeter positional accuracy within a compact field generator tracking volume, making them highly suitable for minimally invasive interventions. Similarly, Polhemus [75] provides a versatile range of 6-DoF tracking solutions, including micro sensors designed to be embedded in surgical tools within various field generator configurations. Their systems are valued for robust tracking without line-of-sight and low drift, but may offer less optimized form factors for ultra-small interventional tools compared to Aurora. Aimooe [76] also develops a similar induction principle based system for clinical applications. Amisco [77] provides the manufacturing of medical micro coils, that can be integrated into tracking sensors across different platforms.

In contrast, the devices which is integrated with magnetic sensors use Hall effect or anisotropic magnetoresistive (AMR) sensors to capture magnetic field vectors directly. OMMO [78] have explored this approach, offering compact, low-power, and potentially low-cost alternatives to micro coils systems. This system can be easily manufactured and facilitated the miniaturization of electronics. However, their localization accuracy can be more susceptible to environmental distortion and sensor noise, especially at longer working distances.

**Figure 9:**
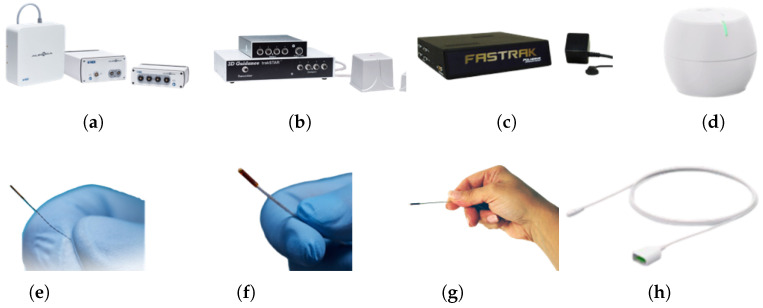
Commercial devices including field generators and micro sensors. (**a**) NDI Inc., Waterloo, ON, Canada: Aurora Field Generator. (**b**) NDI Inc., Waterloo, ON, Canada: 3D Guidance Field Generator. (**c**) Polhemus, Colchester, VT, USA: Fastrak Field Generator. (**d**) OMMO Inc., Carrollton, TX, USA: Orbit Field Generator. (**e**) NDI Inc., Waterloo, ON, Canada: Aurora Sensor. (**f**) NDI Inc., Waterloo, ON, Canada: 3D Guidance Sensor. (**g**) Polhemus, Colchester, VT, USA: Micro Sensor. (**h**) OMMO Inc., Carrollton, TX, USA: Axon Sensor.

An overview of the mentioned commercial devices is shown in Figure 9. All of the EMTS are basically composed of the field generator and tracking sensor. Overall, induction principle based systems are currently employed for high-precision clinical navigation in constrained spaces, while systems that integrate magnetic sensors are gaining traction for wearable, portable, or cost-effective applications, like capsule robots and motion capture systems.

### 5.2. Clinical Application

**Figure 10:**
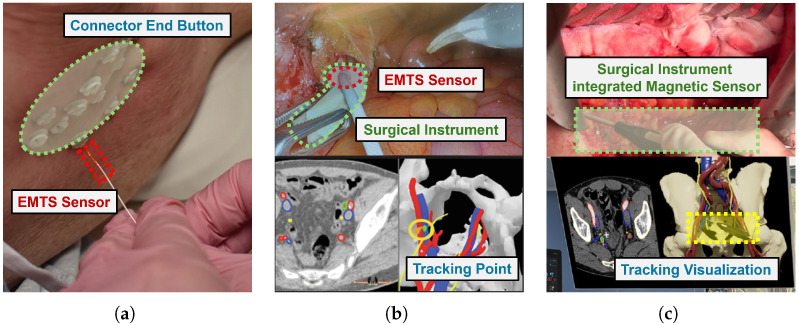
The representative clinical applications. (**a**) Micro coil-based guidewire used for reconstructing implant geometry. (**b**) Image-guided navigation with EMTS during robot-assisted surgery. (**c**) Enhanced CT scan of the patient’s 3D digital model displaying the location of tracked surgical instruments.

EMTS have emerged as a fundamental component of modern surgical navigation, offering high-precision, real-time instrument localization as shown in Figure 10. In a typical clinical workflow, a two- or three-dimensional digital model of the patient’s anatomy is reconstructed from preoperative imaging modalities such as CT, MRI, or other image-guided technologies. This model is subsequently registered to the patient’s position in the operating room, enabling accurate spatial correlation between the model and the patient. Within this framework shown in Figure 10a, EMTS continuously monitors and visualizes the position and orientation of surgical instruments, thereby allowing the surgeon to localize the target point with high precision [79].

EMTS enhanced surgical instrument placement accuracy as shown in Figure 10b, reduced localization uncertainty, and improved treatment reproducibility [80]. In Figure 10c, ETMS is also a possible solution that is not limited by the line-of-sight dependency of optical systems and the increased radiation exposure associated with fluoroscopic guidance [81]. In the field of interstitial brachytherapy, Saurer et al. [34] conducted a systematic review demonstrating that EMTS can improve catheter placement accuracy and workflow efficiency, while also outlining the technological developments required for broader clinical adoption. Furthermore, Agostino et al. [82] evaluated the efficacy and safety of integrating real-time EM tracking with hyperfractionated stereotactic body radiation therapy (SBRT) in patients with intermediate-risk prostate cancer, highlighting its potential to enhance target localization and treatment delivery precision without compromising patient safety.

In dentistry, Gao et al. developed the TianShu-ESNS, an EMTS-guided implant navigation system with a virtual calibration method that achieved sub-2 mm positional accuracy in bot phantom and animal studies [83]. This research eliminated the line-of-sight constraints of optical tracking and reducing intraoperative recalibration requirements. In functional neurosurgery, Burchinel et al. [84] verified that intraoperative EMTS localization could accurately predict final deep brain stimulation (DBS) electrode positions, with mean deviations of approximately 1mm from postoperative imaging, avoiding repeated CT or MRI acuisitions. In cardiovascular interventions, Jia et al. [85] proposed a hybrid echocardiography catheter localization framework that fused NDI EMTS devices with deep learning based segmentation, enhancing tip detection in low-resolution US and avoiding the radiation emission for structural heart procedures.

The integration of EMTS with other novel sensing and imaging technologies has also been developed and used in open and robot-assisted surgeries [86]. Ivashchenko et al. [87] demonstated that combining CBCT-based registration with continuous electromagnetic tracking of the liver surface allowed accurate lesion localization during open liver resections. Similarly, Aguilera Saiz el al. [81] showed that embedding EMTS into a daVinci robotic platform for sentinel lymph node biopsy ahieved a 91% identification rate of predefined nodes. This research confirmed the feasibility and safety for image-guided robotic urologic oncology. In orthopaedic trauma, Xia et al. [88] reported that Kalman filter based fusion of EMTS and optical tracking enabled robust bone needle navigation in pelvic fracture reduction, and maintained the precision during temporary occlusions or magnetic field distortion.

Beyond navigation accuracy, recent clinical application also attract on how to improve the procedural efficiency and reducing morbidity. In hepatobiliary surgery, Zhu et al. [89] achieved a 94.7% puncture success rate for EMTS-assisted ultrasound-guided percutaneous transhepatic one-step biliary fistulation. In endovascular roboitcs, Li et al. [90] integrated flexible electromagnetic guidewire shape sensing with vascular ultrasound in a multirobot magnetic catheter system, enabling real-time 3D reconstruction of both device geometry and vasculature without radiation emission. Zhang et al. [91] developed a Maxwell coil based gradient magnetic field generator integrated with an inertial measurement unit to provide sub-millimeter localization accuracy for surgical instruments. Dürrbeck et al. [38] addressed the challenge of patient-induced motion artefacts in breast interstitial brachytherapy. This research showed that external reference sensors could effectively compensate for disturbance and reduced the mean positional error to 0.11mm below the intrinsic EMTS precision.

These clinical results collectively illustrate that EMTS combined with other complementary modalities can extend the precise and spatial feedback into anatomical regions and procedural contexts where traditional optical or fluoscopic methods are limited.

### 5.3. Comparison

There are many studies related to assessing the EMTS and can be found in the literature, e.g., refs. [92,93,94,95,96]. However, the results of these studies are not comparable because different measurement protocols and evaluaition methods were used. Different accuracy forms are used to express erros and noise. Thus, an evaluation of an implantable electromagnetic micro sensor for computer-assisted surgery is introduced to give the standard comparison index [42].

The feature comparison of different categorized EMTS is shown as the Table 1. A comparative analysis of magnetic localization technologies reveals trade-offs between the tracking accuracy and sensor size. Induction coils offer high AC sensitivity and miniaturization for interventional tracking but cannot measure static fields. Integrated magnetic sensors provide various measurement capabilities and low-cost integration, but are limited by noise and drift. Magnetometric cameras deliver higher sensitivity but require complex, costly systems often needing magnetic shielding. The optimal choice is thus application-specific, hinging on the required precision, measurement type, and practical constraints.

**Figure 11:**
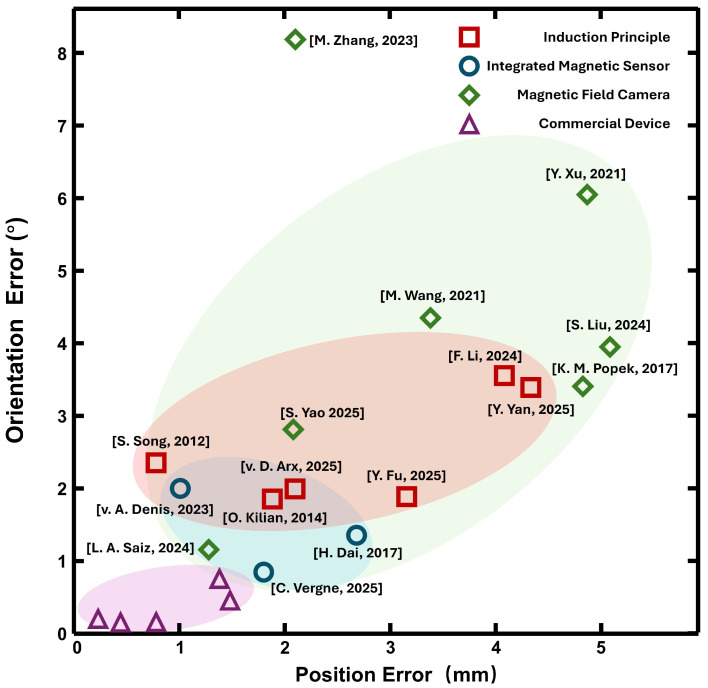
Performance of various research studies and commercial devices shown based on position RMS error and orientation RMS error [29,30,35,36,39,48,50,52,54,56,58,59,62,65,66,67,99].

An intuitive overview of the study results of EMTS is given by Table 2 and Figure 11. The root mean square (RMS) error is commonly used to evaluate the position error for EMTS [97]. In this figure, the performance of different EMTS is compared by plotting position error on the x-axis and orientation error on the y-axis, as shown in Figure 11. The performance data is mentioned in their studies and not consider different measurement protocols. The majority of the standardized studies used one of the most common devices, the NDI Aurora and 3D Guidance on different clinical surgeries. Concerning precision, most assessments show promising results below 2.0 mm. For some studies, their ke y contribution might be innovative mechanical design [52], cost-effective system design [98], and novel magnetic localization technique [66].

**Table 2:** Physical properties and performance of commercial EMTS. N/A means the data is not available.

Field Generator			Sensor			Accuracy	
Type	Shape	Work Space/mm	Size/mm	Degree of Freedom	Update Rate/Hz	Position/mm	Orientation/°
Aurora [100]							
Planar FG	Cube	500 × 500 × 500	ϕ0.3 × 2.5	5	40	0.70	0.20
	Dome	660	ϕ0.3 × 2.5	5	40	1.10	0.20
Planar FG	Cube	500 × 500 × 500	ϕ1.8 × 9	6	40	0.48	0.30
	Dome	660	ϕ1.8 × 9	6	40	0.70	0.30
Tabletop FG	Dome	600	ϕ0.3 × 2.5	5	40	1.20	0.50
	Dome	600	ϕ1.8 × 9	6	40	0.80	0.70°
Window FG	Cylinder	250	ϕ0.3 × 2.5	5	40	N/A	N/A
	Dome	600	ϕ0.3 × 2.5	5	40	N/A	N/A
Window FG	Cylinder	250	ϕ1.8 × 9	6	40	N/A	N/A
	Dome	600	ϕ1.8 × 9	6	40	N/A	N/A
3D Guidance [101]							
Mid-Range	Cube	560 × 460 × 600	ϕ0.56 × 12 ϕ0.9 × 7.25 ϕ1.5 × 7.7 ϕ2.0 × 9.9 7.9 × 8 × 19.8	6	80	1.40	0.50°
Short-Range	Cube	560 × 460 × 600	ϕ0.56 × 12 ϕ0.9 × 7.25 ϕ1.5 × 7.7 ϕ2.0 × 9.9	6	80	N/A	N/A
Polhemus [75]							
Viper	N/A	1820	ϕ1.8	6	240	0.38 (static)	0.10° (static)
Fastrak	N/A	N/A	ϕ1.8	6	120	0.76 (static)	0.15° (static)
Patriot	N/A	N/A	ϕ1.8	6	60	1.52 (static)	0.40° (static)
LIBERTY	N/A	N/A	ϕ1.8	6	240	0.76 (static)	0.15° (static)
G4	N/A	N/A	ϕ1.8	6	120	2.0 (static)	0.50° (static)
Aimooe [76]							
Magpilot	Cube	300 × 300 × 300	ϕ0.45 × 8.0 ϕ0.45 × 5.0 ϕ1.5 × 10.0	5 5 6	80	2.0	N/A
OMMO [78]							
Orbit + Axon	Sphere	650	21.20 × 5.30 × 4.10 26.30 × 5.30 × 4.10	5 6	N/A	0.2 0.17	0.10° 0.08°

The purple region represents commercial systems with position errors typically below 1.5 mm and orientation errors under 1°. This cluster reflects the high precision and clinical reliability achieved through long-term optimization of sensor design and stable electromagnetic field generation. The red region corresponds to induction principle based systems reported in research studies and clinical applications. These systems generally achieve position errors between 1 mm and 4 mm and orientation errors ranging from 1° to 3°. While they demonstrate promising accuracy, particularly in controlled laboratory settings. The blue region and green region illustrates research systems employing the magnetic sensor and the magnetic field camera, respectively. Their performance distribution is broader, with some systems approaching commercial-grade accuracy, while others exhibit position errors exceeding 5 mm and orientation errors reaching up to 8°. This variability can be largely attributed to differences in sensor resolution, array configuration, and distortion correction strategies. The observed spread also highlights the need for standardized evaluation metrics to enable fair performance comparisons under consistent testing conditions.

This table summarizes the key specifications and performance metrics of representative EMTS from major commercial and emerging companies, including Northern Digital Inc., Polhemus, Aimooe, and OMMO. The table lists the field generator configuration, workspace dimensions, sensor size, degrees of freedom, update rate, and both position and orientation accuracy. The Aurora platform offers multiple field generator geometries and workspaces. Miniaturized 5-DoF sensors achieve a position error down to 0.7 mm and an orientation error lower to 0.2°. This specification makes them suitable for integration into fine interventional tools. The 3D Guidance provides a larger mid-range and short-range cube workspace with 6-DoF sensors of varying diameters. This system offers up to 1.4 mm position error and 0.5° orientational error at an 80 Hz update rate. Polhemus systems are characterized by high update rates and compact 6-DoF sensors. Its static position errors can be as low as 0.15 to 0.38 mm and orientation erros between 0.10° and 0.50°, depending on the sensors. In the emerging company, Aimooe’s Magpilot offers a cube workspace with 5-DoF and 6-DoF sensors, and a position error near 2 mm. OMMO’s Orbit and Axon employs a spherical workspace with 5-DoF sensor and achieves sub-millimeter accuracy (0.17 to 0.20 mm) and orientation errors around 0.10°. The figure and table illustrate both the state-of-the-art performance envelope in EMTS technology. The trade-offs between workspace size, sensor miniaturization, and accuracy requirements need to be considered for clinical applications.

Over the past five years, EMTS accuracy has reached a clinically serviceable level, prompting a rapid expansion of the clinical applications. In particular, commercial systems generally deliver more consistent tracking performance than research prototypes. Nevertheless, clinical translation remains constrained by substantial barriers because the tracking performance cannot be guaranteed across all clinical contexts. Current researches increasingly emphasize multimodal sensor fusion and adaptive distortion compensation to improve robustness. Equally important are calibration and standardized evaluation metrics to ensure reproducibility across institutions. Looking ahead, the research article should first strengthen EMTS robustness under realistic clinical constraints. Regulatory approval must be supported by rigorous evidence. At the same time, EMTS should be integrated into routine workflows to reduce the cognitive load and operational effort. These goals should be achieved while maintaining a favorable cost–performance balance to enable adoption at scale.

## 6. Discussion

**Figure 12:**
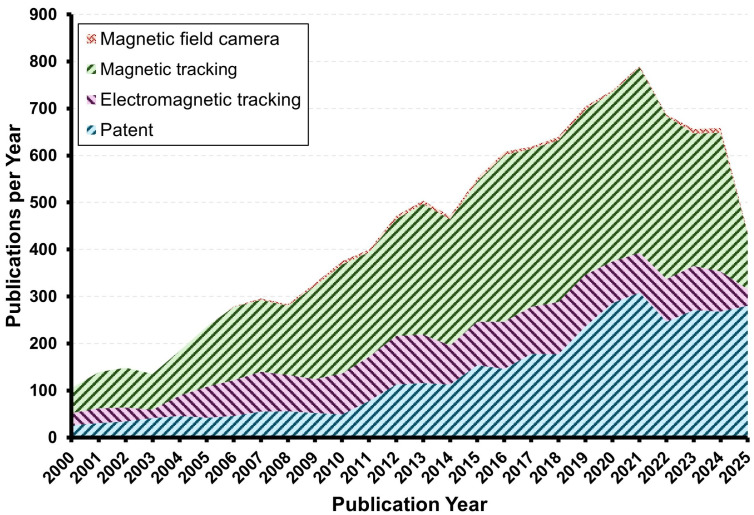
Publications per year from research articles (electromagnetic tracking, magnetic track, and magnetic field camera) and patents.

As shown in Figure 12, the number of publications related to EMTS has increased steadily over the past two decades. The figure illustrates annual publications across different tracking methods as well as patents. Since around 2010, the number of patents has risen sharply, reflecting the rapid industrialization and commercialization of EMTS technology. At the same time, research articles on magnetic tracking and electromagnetic tracking have continued to grow, demonstrating the strong academic interest in advancing the precision, robustness, and clinical applicability of these systems. This trend indicates that EMTS is moving beyond the laboratory and becoming a focus of translational research aimed at clinical adoption. Despite substantial advancements, several challenges persist in the development and clinical application of EMTS:

1. Simultaneous localization and navigation: Future research should focus on developing the simultaneous localization and navigation system with high accuracy performance. In some areas where expensive image-guided modalities are not available, some difficult minimally invasive surgeries can be performed with the convenience of EMTS.
2. Sensor Fusion: EMTS integrated with other image-guided modalities could enable high-accuracy localization and trajectory guidance. This will require advanced sensor fusion strategies and algorithms to maintain robustness in anatomically complex and dynamic surgical environments.
3. Distortion compensate algorithms: There remains a critical requirement for adaptive, real-time algorithms to compensate magnetic field distortions induced by external disturbances. Emerging approaches enhance localization accuracy and robustness, including both hardware and software solutions.
4. A standardized evaluation protocol: A standardized evaluation protocol should incorporate well-defined test phantoms, consistent reference frames, and harmonized performance metrics to enable reproducible benchmarking across systems and institutions. Establishing such elements would not only facilitate objective comparison but also accelerate regulatory approval and clinical translation of EMTS.
5. Cost-effective: Currently, the overall cost of EMTS remains high. Achieving economic viability will require the optimization of hardware architectures and sensor designs while maintaining performance standards. Balancing performance and affordability is critical to facilitate widespread implementation across diverse clinical scenarios.

## 7. Conclusions

EMTS have evolved into a versatile and powerful navigation technology for minimally invasive and image-guided interventions [102,103]. Over the past five years, significant progress has been made in both macro and micro scales, including the system designs, sensor optimization, and integration with other guidance modalities. Commercial devices currently are focusing on accuracy and reliability, while research studies are rapidly improving the tracking performance and expanding the application. Comparative analyses indicate that the induction principle based systems excel in high tracking precision and work well in constrained-space navigation. Moreover, the magnetically integrated sensor arrays offer advantages in portability. Looking forward, the further adoption of EMTS in routine clinical workflows will depend on advances in distortion algorithms, standardized accuracy assessment protocols, and the development of compact, robust systems adaptable to varied surgical environments. (EMTS is poised to play an increasingly central role in enhancing procedural precision, reducing radiation exposure, and improving patient outcomes across a broad range of medical specialties).

## Figures and Tables

**Table 1:** Feature comparison of different categorized EMTSs.

Feature	Induction Principle	Integrated Magnetic Sensors	Magnetic Field Camera
Advantages	High sensitivity to AC fields. Mature technology and low cost. Miniaturization on intervention.	Measures both DC and AC fields. Low cost and easy integration. Small size and low power consumption.	High sensitivity and accuracy. Measures absolute magnetic field value. Wide tracking workspace.
Disadvantages	Incapable of measuring static fields. Orientation and pose calculation are complex. Susceptible to ambient AC noise.	Limited sensitivity, and temperature drift. Calibration for non-linearity and drift. Often requires magnetic flux concentrators.	System complexity and high cost. Mutual electromagnetic influence. All disadvantages from Integrated Magnetic Sensors.

## Data Availability

No new data were created or analyzed in this study.

## Abbreviations

The following abbreviations are used in this manuscript:

EMTS	Electromagnetic Tracking System
MIS	Minimally invasive surgery
CT	Computed tomography
MRI	Magnetic Resonance Imaging
US	Ultrasound
OCT	Optical Coherence Tomography
IVUS	Intravascular Ultrasound
